# Predictability of orthodontic tooth movement with aligners: effect of treatment design

**DOI:** 10.1186/s40510-022-00453-0

**Published:** 2023-01-16

**Authors:** Tommaso Castroflorio, Ambra Sedran, Simone Parrini, Francesco Garino, Matteo Reverdito, Riccardo Capuozzo, Sabrina Mutinelli, Simonas Grybauskas, Mantas Vaitiekūnas, Andrea Deregibus

**Affiliations:** 1 Department of Surgical Sciences, Dental School of the University of Torino, Via Nizza 230, 10126 Turin, Italy; 2 Poggibonsi, Italy; 3 Turin, Italy; 4 Cuneo, Italy; 5 Caserta, Italy; 6 Department of Neuroscience, School of Dentistry, Section of Pedodontics, University of Padova, Via VII Febbraio 2, 35122 Padua, Italy; 7 University of Ferrara, Vilnius, Lithuania; 8 Kaunas University of Technology, Kaunas, Lithuania

**Keywords:** Clear aligners, Orthodontic tooth movement, Attachments, Treatment design

## Abstract

**Backgrounds:**

The present study was designed to define: (1) which are the less predictable OTM with Invisalign aligners when the treatment plan is designed by expert operators, (2) if the presence and shape of attachments influence the predictability of OTM and (3) if patients’ demographics influence OTM predictability. The sample comprises 79 prospectively recruited patients (mean age 30.8 years; SD 12.0; 23 M, 56 F), treated by expert operators with an average of 27 aligners (SD 15) in the maxillary arch and 25 aligners (SD 11) in the mandibular arch. Post-treatment digital models and final virtual treatment plan models were exported from ClinCheck^®^ software as STL files and subsequently imported into Geomagic Qualify ^®^software, to compare final teeth positions. The differences were calculated and tested for statistical significance for each tooth in the mesial–distal, vestibular–lingual and occlusal–gingival directions, as well as for angulation, inclination and rotation. In addition, the statistical significance of categorical variables was tested.

**Results:**

The lack of correction was significant for all movements and in all group of teeth (*P* < 0.01) except for the rotation of maxillary first molar. The prescribed OTM, the group of teeth and movement, the frequency of aligner change and the use of attachment influence the outcome. The greatest discrepancies in predicted and achieved tooth position were found for angular movements and rotation of teeth characterized by round-shaped crowns, for a ratio of approximately 0.4° per 1° prescribed. Optimized attachments for upper canines and lower premolar rotation seem not working properly. Second molar movements are mostly unexpressed. Furthermore, changing the aligner every 14 days will reduce the lack of correction of the 12% with respect to 7 days aligner change.

**Conclusions:**

Predictability of orthodontic movement with aligners still has limitations related to the biomechanics of the system: the shape of some attachments and the characteristics of aligner material need to be redefined. However, the results of this study allow to properly design the virtual treatment plan, revealing how much overcorrection is needed and which attachments are most effective.

## Background

Over the past two decades, the orthodontics’ field has been revolutionized by technological advances. Three-dimensional imaging and intraoral scanners have expanded both diagnostic and treatment planning capabilities and digital fabrication of appliances [1–3]. Together with the increased adult patient demand for orthodontic treatment [4–6] and the push toward personalized treatment, these developments resulted in a growing demand for clear aligners, to the point that they are now an option of any orthodontic practice [7]. However, literature shows that the reliability of orthodontic tooth movement (OTM) with those devices such as Invisalign^®^ aligners (Align Technology, San José, CA, USA) seems not encouraging.

Several papers have demonstrated that what is virtually planned is not what is clinically achievable [8–12]. Even if some limitations in the appliance system remain, it should be considered that clear aligner orthodontics techniques are customized not only for the patients but for orthodontists too [13]. Therefore, virtual treatment plan design, in terms of attachments’ design and placement [14–18], OTM staging [19–21] and aligner deformation overengineering [13, 22, 23], or in other words aligners biomechanics knowledge, plays a crucial role in defining the quality of the orthodontic treatment with aligners. It should be noted that aligners biomechanics knowledge is not necessarily proportionally to the number of started treatments: this is an important consideration to remember for investigators collecting cases from private practitioners and for whom clinical preferences are unknown [10].

Despite these considerations, Invisalign system seems not completely effective in reaching treatment objectives since there are several factors connected to appliance characteristics as well as patients and orthodontist performance which could affect the treatment achievement. However, to our knowledge, most of the published studies comprised small groups of patients [13, 20, 24] and sometimes the sample was not homogeneous, including both adults and adolescents [8]. Therefore, additional studies are needed to confirm or confute published results.

Based on these premises, the present study was designed to assess the effectiveness of Invisalign^®^ treatment in achieving the prescribed movements in a group of patients larger than those of the previous studies. Patients’ baseline and treatment conditions as well as appliance features were analyzed to define their influence onto the predictability of OTM. This prospective multicenter study was conducted to verify the hypothesis that clear aligner treatment does not completely fulfill the pretreatment goals at the end of the first set of aligners.

## Materials and methods

### Study design and patient selection

The sample comprised 79 patients (mean age, 30.8 years; SD, 12.0), of which 23 were men. The patients were treated for 9.8 months (SD, 3.8) on both arches, with an average of 27 clear aligners (SD, 15) in the maxillary arch and 25 clear aligners (SD, 11) in the mandibular arch.

Subjects were recruited prospectively at the Department of Orthodontics of the University of Turin, which was the coordinating center, and at five private Italian orthodontics offices. All co-investigating orthodontists have recognized clinical and teaching skills. In fact, practitioners completed an enrollment questionnaire to joining the trial, which collected information on the practitioners and their practices. The inclusion criteria for practitioners were as follows: certified orthodontist with huge and renewed experience in Invisalign treatments; with the ability to collect intraoral scans and upload (via internet) the files obtained to a central repository; affirming that the practice can devote sufficient time in patient scheduling to allow focused recording of all data required for the study; and does not anticipate retiring, selling the practice or moving during the study [25]. Signed, written informed consent was required before inclusion in the trial.

The five selected orthodontists had a mean age of 45.6 years (SD, 8.2) at the beginning of the study.

Patients were selected accordingly to the following inclusion criteria: complete permanent dentition, with the exception of third molars; Invisalign aligner treatment on both arches; active tooth movements programmed at the standard rate recommended by the Align Tech technician; no intermediate corrections or additional aligners; aligners change every 7 to 14 days. Exclusion criteria were the need for oral surgery or dental restorations, and for combo treatments (i.e., combination of aligners with any other orthodontic appliance); reported previous orthodontic treatment; presence of prosthetic restorations and/or periodontal problems; signs and/or symptoms of temporomandibular disorders. All participants included in this prospective observational study had Class I or mild Class II malocclusion with mild-to-moderate crowding or spacing in the maxillary and mandibular dental arches (non-extraction cases). Chewies to improve aligner seating and intermaxillary elastics were not used. Interproximal enamel reduction was performed as prescribed in each patient's virtual treatment plan.

Regarding treatment achievement, the real post-treatment.stl file, obtained by the final intraoral scan, was overlapped to the planned post-treatment.stl file, exported from the virtual setup. This procedure was repeated for each patient. Thus, a total of 2212 teeth were measured in the entire sample.

Ethics approval was obtained from the Research Ethics Board (Città della Salute e della Scienza di Torino #157/2020), and informed consent was acquired from each subject before entering the study.

The study protocol was registered on ClinicalTrials.gov (#NCT05356780).

Control appointments were fixed at 6-week interval in both the University and the private settings. At the delivery appointment, patients were instructed to wear their aligners for 22 h per day. Patients understood that they were part of a research study, and honest reporting of their compliance was critical. Compliance was also verbally confirmed at each appointment.

### Measurement of predicted and obtained orthodontic tooth movement

Digital models were exported from the ClinCheck^®^ software (Align Technology, San José, CA, USA) as stereolithography files. Final stage.stl files were labeled as “predicted outcome.” Stereolithography files were also obtained from the intraoral scans of the “refinement” stage or of the retention stage and labeled as “achieved outcome” since they represented the actual outcome after the first set of aligners [26].

All.stl files were deidentified, and soft tissues were digitally removed to ensure that the evaluation was based solely on tooth surface characteristics. The superimposition of the post-treatment.stl file (achieved outcome) on the planned final stage.stl file (predicted outcome) was performed using Geomagic^®^ Qualify software (3D Systems, Rock Hill, SC, USA).

**Fig. 1:**
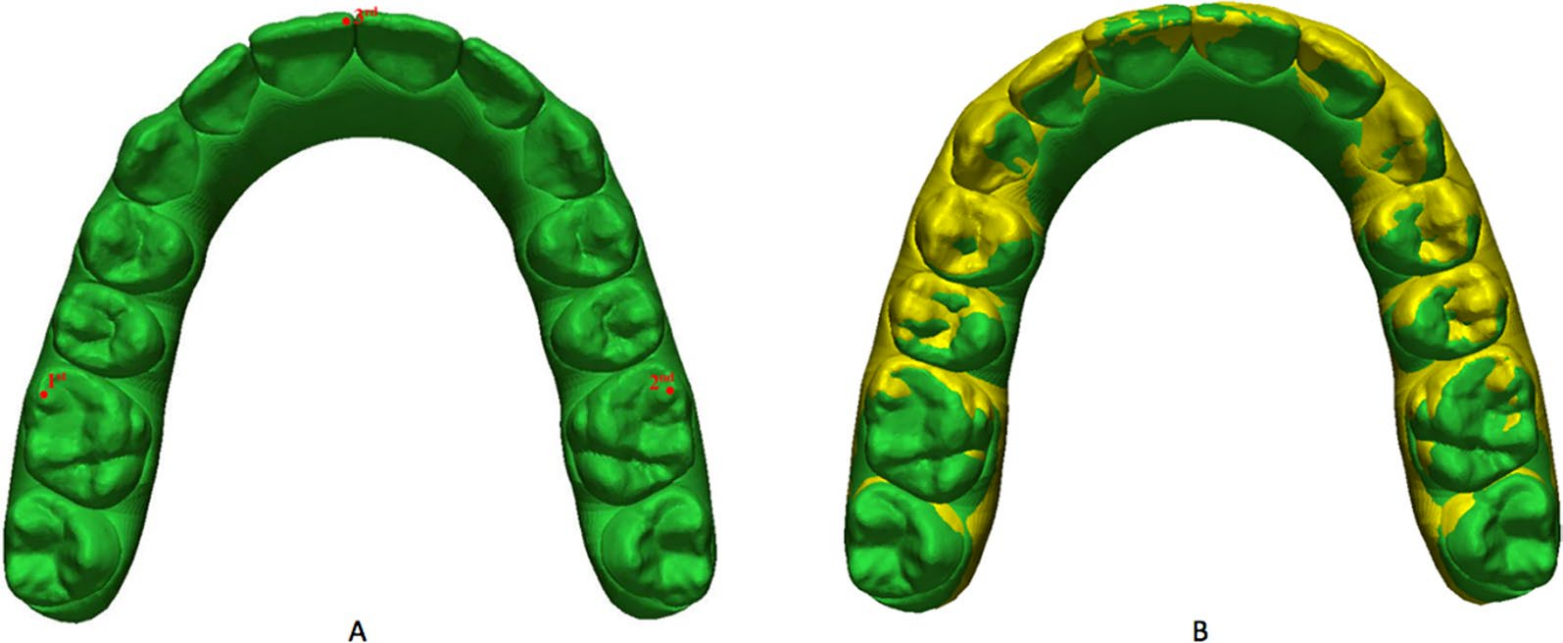
**A** Overlay performed by the landmark-based method; **B** Overlay performed by the surface-based method (best-fit alignment) using a best-fit algorithm

The dental arches were superimposed using the landmark-based method. The three anatomical landmarks were: the mesio-vestibular cusps of the first molars (1–2) and the mesial-incisal point of the right central incisor (3). The results of the overlay performed with the landmark-based method are presented in Fig. 1. Superimposition accuracy was increased by the surface-based method (best-fit alignment) using a best-fit algorithm [27].

**Fig. 2:**
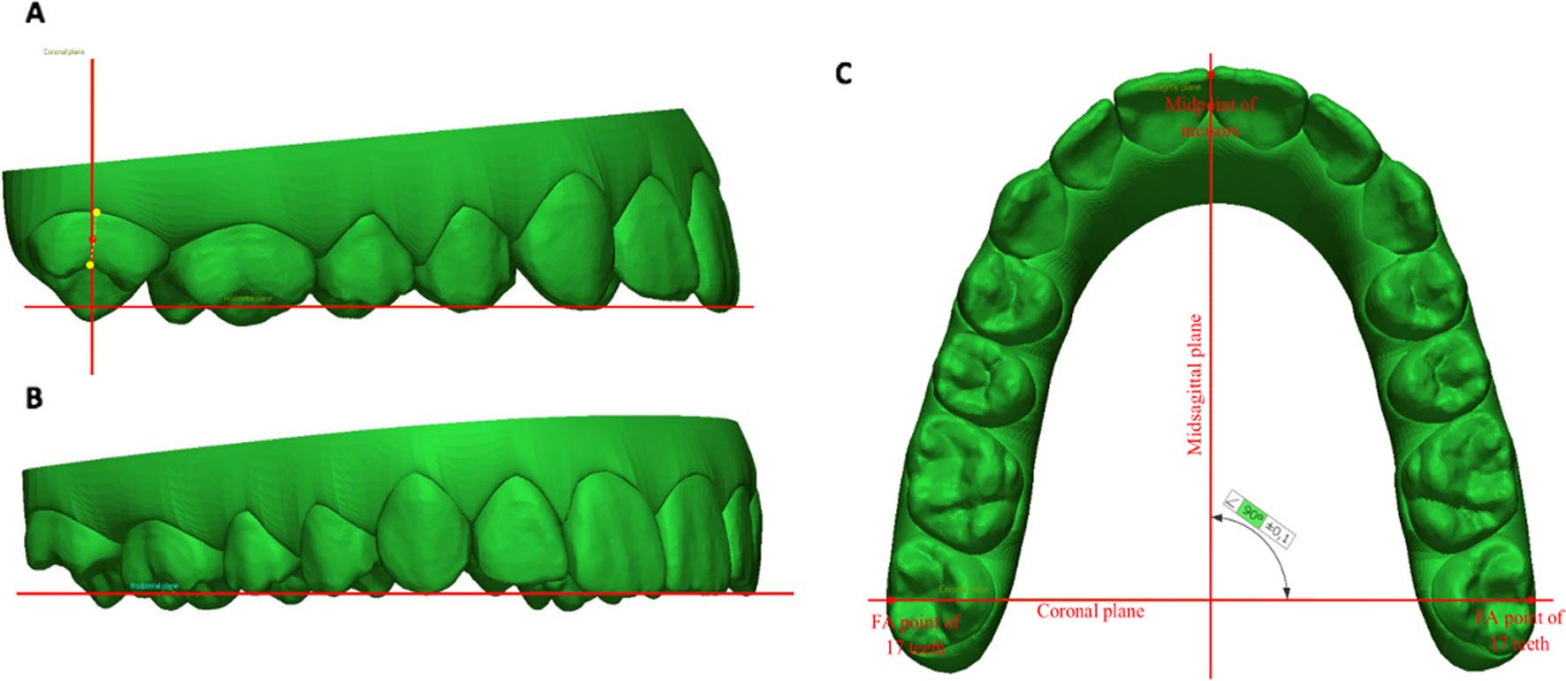
Coronal (**A**), occlusal (**B**), median (**C**) reference planes

Therefore, three reference planes were identified on the virtual treatment plan model (Fig. 2). The occlusal plane was created considering the midpoint of the right and left incisal edges and the tips of the mesio-buccal cusps of the right and left first molar [28]. The coronal plane passes through a midpoint between the facial axis (FA) points of teeth 17 and 27 and the midpoint of the right and left incisal edges. The coronal plane is perpendicular to the occlusal plane. The median plane passes through a midpoint between the incisors and is perpendicular to the occlusal and coronal planes [29].

The facial axis of the clinical crown (FACC) and FA points [30] were then placed on the post-treatment model too. The post-treatment model was segmented to isolate each tooth as a separate object. The software then superimposed each tooth from the segmented post-treatment model on the corresponding tooth of the non-segmented virtual treatment model using the best-fit surface-based algorithm. Finally, the differences between the achieved and predicted position of each tooth were calculated.

The following variables were considered for the analysis: angulation (mesial or distal tip), inclination (in–out, measured as the angle between the occlusal plane and a tangent plane passing through the FA point [30]), rotation (the rotation of each tooth was measured between the vector, which was created through the mesial and distal points, and the median plane), mesio-distal movement (distance between FA points and the coronal plane), vertical movement (distance between the FA points and the occlusal plane), and buccal/lingual movement (distance between the FA points and the median plane).

Because the software allows for differences that are too small to be clinically relevant, the threshold values were chosen with reference to the American Board of Orthodontics (ABO) model grading system for case evaluation [31]. According to the criteria of the “model grading system,” discrepancies of 0.5 mm or greater in the alignment of the contact points and marginal ridges result in point subtraction. A marginal ridge discrepancy of 0.5 mm is equivalent to a crown tip deviation of 2° for an average-sized molar. Therefore, differences of 0.5 mm or more in the mesial–distal, bucco-lingual and occlusal–gingival directions and differences of 2° or more in tip, torque and rotation were considered clinically relevant [13].

### Statistical analysis

The lack of correction (LC), or the difference between the prescribed result and the achieved correction, represents the primary outcome of the study, while the amount of prescribed movement (PM), data extracted from the Invisalign website of each orthodontist and for each patient, constitutes the primary exposure. Both LC and PM represent continuous variables measured in millimeters, for linear measurements, and degrees, for angles. Another variable, type of movement (TM), identifies the movement associated to PM and LC. This comprised six categories such as angulation, inclination, rotation, and mesio-distal-, vertical- and bucco-lingual movements.

Regarding the categorical variable teeth, this was included in the datasets codifying each single type of tooth as a category, therefore 28 in total. Then, a new multi-level variable, teeth group (TG) was generated from the previous one including each tooth into a category of teeth with similar characteristics (maxillary arch: central incisors, lateral incisors, cuspids, bicuspids, first molars and second molars; mandibular arch: incisors, cuspids, bicuspids, first molars and second molars).

In addition, for each patient and for each TG, the mean values of LC and PM were estimated, averaging the corresponding values of the teeth included in the same category. Those means were incorporated in a new dataset as two variables mLC (LC means) and mPM (PM means) together with the variable TG and the patients’ identification.

The other tested predictors were age (in years), treatment time (in months), frequency of aligner change (every 7 days, 10 days or 14 days) and the three-level categorical “attachment” variable (none, conventional and optimized).

#### Specific aim #1

The first specific aim of the study was to establish whether the final position of the teeth achieved after the treatment was equal to that one of the virtual model obtained in the ClinCheck^®^. In other words, the null hypothesis stated that the variable mean lack of correction (mLC) was equal to zero. Before starting the analysis, we conducted a logarithmic transformation of the data to normalize the distribution. In fact, mLC was not normally distributed since the graph did not show a bell shape as well as the Shapiro–Wilk test was significant (*P* < 0.001). After that, to test the null hypothesis, we run a one-sample t test for each type of movement for every single group of teeth. The descriptive statistics of mLC and mPM stratified for group of teeth and movements has been summarized as median and interquartile range.

#### Specific aim #2

The second specific aim of the study was to address the question if the primary outcome LC was affected from some predictors such as the pretreatment prescription, the moved teeth, treatment duration, the employment of attachments and the frequency of aligner change as well as the patients’ age and sex. To achieve the aim, we built a multiple linear regression model using the cluster option, since we wanted to indicate that the observations were clustered into patients. Consequently, the correlation among the tooth movements belonging to the same patient was allowed. In addition, to perform a cautious analysis, the Huber–White sandwich estimator was used to obtain robust standard errors.

The primary outcome LC and the predictor PM were included in the dataset as log-transformed variables. In fact, PM as well as LC did not follow a normal distribution (Shapiro–Wilk test, *P* < 0.001). In addition, PM and LC comprised the observations derived from all types of movements, which were interpreted as units of movement and not as linear or angular value.

During the model building procedure, the other covariates were step-forward tested: frequency of aligners changes (every 7 days, 10 days or 14 days) and the three-level categorical variable “attachment” (none, conventional and optimized), along with age, sex and treatment time. When the result was nonsignificant, the variable was excluded from the final model. This was the case of the last three predictors or age, sex and treatment time.

The model goodness of fit was estimated by means of the coefficient of determination (*R*^2^). Afterward, we checked that the key assumptions of multiple linear regression had been respected. At first, the linear relationship between the outcome and the continuous predictors was tested with scatter plots. Then, the assumption of normality of residuals was confirmed graphing a standardized normal probability plot and collinearity was excluded after having estimated the Variance Inflation Factor (VIF; mean, 1.25). Finally, homoscedasticity was examined with the plot of standardized residuals versus predicted values. The *α*-level was fixed at 0.05. All data were analyzed using STATA 14.2 (StataCorp LP, College Station, Tex).

#### Sample size and reliability of the measurements

The sample size of the study was estimated a priori assuming an average lack of correction of 50%, or a difference of 0.5, as reported in the paper of Haouili et al. [8], a power of the test of 90% and an *α*-level of 0.05 (one-sample means t test). The standard deviation was assumed as 1. Under those conditions, the sample size amounted to 43 patients.

The reliability of the measurements was assessed using the intraclass correlation coefficient (ICC). The operator (MV) who performed all digital measurements repeated 20 measurements twice for each one of the six movements prescribed (240 measurements in total), with a 21-day interval between the two estimations. The ICC amounted to 0.99, showing an excellent agreement between the repeated measurements.

## Results

### Specific aim #1

The amount of mLC was significant for all movements and in all group of teeth (*P* < 0.01) except for the rotation of maxillary first molar (median, 1.47; Iqr, 1.21; one-sample *t* test conducted on logarithmic transformed data; *P* = 0.3613). In addition, the maxillary second molar showed a mLC in rotation close to the significance level (median, 1.71; Iqr, 1.52; one-sample *t* test conducted on logarithmic transformed data; *P* = 0.0527). As a consequence, the null hypothesis cannot be rejected, and the teeth did not achieve the prescribed final position.

The median and interquartile range (Iqr) of mPM and mLC variables stratified for type of movement and group of teeth are displayed in Table 1.

**Table 1:** Descriptive statistics (median and interquartile range, Iqr) of the variables prescribed movement (mPM) and lack of correction (mLC), stratified for type of teeth and movements

	Angulation (degree)		Inclination (degree)		Rotation (degree)		Mesio-distal translation (mm)		Vertical translation (mm)		Buccal/lingual translation (mm)	
	mPM	mLC	mPM	mLC	mPM	mLC	mPM	mLC	mPM	mLC	mPM	mLC
*Maxilla*												
C incisors (*n* = 158)	2.70 (1.72)	2.08 (1.19)	5.13 (3.96)	3.53 (2.47)	7.33 (6.03)	2.77 (2.27)	0.56 (0.50)	0.34 (0.40)	0.76 (0.63)	0.31 (0.26)	1.07 (0.82)	0.14 (0.10)
L incisors (*n* = 158)	3.55 (2.53)	2.72 (1.95)	3.99 (2.90)	3.97 (2.46)	10.37 (6.71)	3.91 (2.94)	0.77 (0.75)	0.22 (0.15)	0.67 (0.50)	0.32 (0.25)	0.86 (0.64)	0.20 (0.15)
Canines (*n* = 158)	3.33 (2.30)	3.11 (2.31)	2.99 (1.67)	3.03 (1.86)	11.17 (7.98)	4.66 (3.41)	0.77 (0.91)	0.27 (0.23)	0.62 (0.44)	0.40 (0.36)	0.75 (0.48)	0.32 (0.22)
Premolars (*n* = 316)	3.12 (1.99)	2.50 (1.65)	3.51 (2.49)	4.86 (2.74)	5.52 (3.33)	2.20 (1.41)	0.55 (0.72)	0.25 (0.23)	0.34 (0.27)	0.46 (0.29)	0.97 (0.67)	0.32 (0.24)
1st molars (n = 158)	1.61 (1.63)	1.84 (1.59)	2.12 (1.81)	3.93 (2.69)	4.42 (3.72)	1.47 (1.21)	0.49 (0.74)	0.21 (0.26)	0.26 (0.31)	0.31 (0.24)	0.70 (0.61)	0.31 (0.24)
2nd molars (*n* = 158)	1.66 (3.34)	2.43 (3.07)	1.72 (1.95)	4.17 (3.52)	2.64 (3.25)	1.71 (1.52)	0.45 (0.78)	0.21 (0.20)	0.31 (0.44)	0.32 (0.34)	0.35 (0.41)	0.36 (0.22)
*Mandible*												
Incisors (*n* = 316)	3.03 (1.56)	2.40 (1.32)	5.08 (3.37)	2.84 (2.17)	9.18 (4.71)	2.60 (1.90)	0.61 (0.48)	0.19 (0.14)	1.14 (0.80)	0.34 (0.27)	1.30 (0.92)	0.16 (0.10)
Canines (*n* = 158)	4.99 (3.28)	3.41 (2.68)	3.23 (2.25)	2.90 (2.02)	14.83 (9.38)	4.80 (3.70)	0.50 (0.46)	0.24 (0.21)	0.73 (0.58)	0.33 (0.23)	0.91 (0.72)	0.24 (0.20)
Premolars (*n* = 316)	4.60 (2.52)	3.54 (2.25)	4.09 (2.34)	4.39 (2.46)	8.24 (4.18)	3.84 (2.38)	0.33 (0.34)	0.31 (0.21)	0.36 (0.23)	0.44 (0.26)	0.93 (0.67)	0.27 (0.19)
1st molars (*n* = 158)	1.21 (1.37)	2.35 (2.24)	1.84 (1.82)	4.68 (2.90)	3.08 (3.16)	1.99 (1.57)	0.21 (0.31)	0.27 (0.28)	0.26 (0.29)	0.43 (0.27)	0.65 (0.58)	0.46 (0.45)
2nd molars (*n* = 158)	1.60 (2.53)	2.37 (1.79)	1.46 (2.30)	4.71 (3.51)	2.87 (3.33)	2.28 (2.06)	0.17 (0.27)	0.28 (0.24)	0.28 (0.39)	0.27 (0.20)	0.35 (0.44)	0.59 (0.43)

*Iqr is Interquartile range

All the variable LC (difference between the tooth position achieved after the treatment and that one of the virtual model of the ClinCheck^®^) are significant for all types of movements (one-sample* t* test conducted on logarithmic transformed data; *P* < 0.001), except for the rotation of maxillary 1st molar (one-sample *t* test conducted on logarithmic transformed data; *P* = 0.3613). The difference in rotation of the maxillary 2nd molar was close to the significance level (one-sample *t* test conducted on logarithmic transformed data; *P* = 0.0527)

### Specific aim #2

All predictors included in the regression model reach a significant level (*P* < 0.001). Therefore, the prescribed movement, the group of teeth and movement, the frequency of aligner change and the employment of attachment influence the outcome LC.

In fact, when we consider PM keeping constant the other variables, the multiple linear regression output shows that PM significantly affects LC (*P* < 0.001). In detail, if PM increases by 10%, we expect a 2% increase in LC, and a 9% and 15% when PM rises to 50% and 100%, respectively.

Regarding TM, having the angulation category as reference and fixed the other variables, the highest percent increase in geometric mean of LC is obtained in the inclination, followed in decreasing order by angulation, rotation, vertical, mesio-distal and bucco-lingual translation.

In addition, allowing the variable TG only to vary, mandibular second molars show the highest percent increase in geometric mean of LC from the reference maxillary central incisor. On the contrary, the mandibular incisors present the lowest value below the reference. In sequence we have, from the highest to the lowest LC percentage: mandibular second molars, mandibular first molars, mandibular premolars, maxillary second molars, maxillary canines, maxillary premolars, maxillary lateral incisors, mandibular canines, maxillary first molars, maxillary central incisors and mandibular incisors.

Concerning the frequency of aligner change, the category 14 days only shows a significant difference from the reference 7 days and a decrease in 12% of LC geometric mean (*P* = 0.009), holding the other variables constant. The difference is not significant between the 7-day and 10-day categories.

With respect to the employment of attachments, the difference is significant between the reference (absence of attachment) and use of optimized attachments (LC geometric mean increase, 8%) only when the other predictors are constant. The distribution of attachments is summarized in Table 2.

**Table 2:** Distribution of attachments stratified for group of teeth

	Absence of attachments		Conventional attachments		Optimized attachments	
	Proportion	95% confidence interval	Proportion	95% confidence interval	Proportion	95% confidence interval
*Maxilla*						
Central incisors	0.66	0.63 to 0.69	0.01	0.003 to 0.01	0.33	0.30 to 0.36
Lateral incisors	0.29	0.26 to 0.32	0.10	0.08 to 0.12	0.61	0.58 to 0.64
Canines	0.13	0.11 to 0.15	0.16	0.14 to 0.19	0.71	0.68 to 0.74
Premolars	0.18	0.16 to 0.20	0.15	0.14 to 0.17	0.67	0.64 to 0.69
First molars	0.25	0.23 to 0.28	0.55	0.52 to 0.58	0.19	0.17 to 0.22
Second molars	0.60	0.57 to 0.63	0.32	0.29 to 0.35	0.08	0.06 to 0.10
*Mandible*						
Incisors	0.83	0.81 to 0.85	0.04	0.03 to 0.05	0.13	0.12 to 0.15
Canines	0.25	0.22 to 0.28	0.05	0.04 to 0.07	0.70	0.67 to 0.73
Premolars	0.21	0.19 to 0.23	0.09	0.07 to 0.10	0.70	0.68 to 0.72
First molars	0.42	0.39 to 0.45	0.49	0.46 to 0.52	0.09	0.08 to 0.12
Second molars	0.63	0.60 to 0.66	0.27	0.24 to 0.29	0.10	0.08 to 0.12

However, this linear regression covers the 52% of the dependent variable variance (*R*^2^) only. This means that the predictors partially justify the variability and other unknown relevant conditions should influence LC. As a consequence, the model cannot completely answer the question of specific aim #2. The regression output is displayed in Table 3.

**Table 3:** Multiple linear regression output. The primary outcome lack of correction (LC) and the exposure prescribed movement (PM) were normalized by means a logarithmic transformation

Predictor	Coefficient	Robust standard error	*t*	*P* > *t*	95% confidence interval
Prescribed movement (PM)^*^	0.21	0.02	11.19	< 0.001	0.17 to 0.24
*Movement*^*†*^					
Inclination	0.37	0.03	10.92	< 0.001	0.30 to 0.44
Rotation	–0.13	0.04	–3.17	< 0.001	–0.20 to –0.05
Mesio-distal translation	–2.06	0.07	–31.09	< 0.001	–2.19 to –1.93
Vertical translation	–1.59	0.05	–33.53	< 0.001	–1.69 to –1.50
Bucco-lingual translation	–2.08	0.05	–41.91	< 0.001	–2.18 to –1.98
*Teeth*^*‡*^					
Maxillary lateral incisors	0.13	0.05	2.43	0.02	0.02 to –0.24
Maxillary canines	0.23	0.08	2.90	0.01	0.07 to 0.39
Maxillary premolars	0.19	0.07	2.89	0.01	0.06 to 0.32
Maxillary 1st molars	0.03	0.08	0.43	0.67	–0.12 to 0.19
Maxillary 2nd molars	0.29	0.08	3.70	< 0.001	0.13 to 0.44
Mandibular incisors	–0.14	0.06	–2.20	0.03	–0.27 to –0.01
Mandibular canines	0.08	0.08	1.04	0.30	–0.08 to 0.24
Mandibular premolars	0.29	0.07	4.32	< 0.001	0.16 to 0.43
Mandibular 1st molars	0.32	0.08	4.20	< 0.001	0.17 to 0.47
Mandibular 2nd molars	0.49	0.08	6.11	< 0.001	0.33 to 0.65
*Attachment*^*§*^					
Conventional	0.09	0.06	1.72	0.09	–0.02 to 0.20
Optimized	0.08	0.03	2.27	0.03	0.01 to 0.15
Frequency of aligner change^**^					
10 days	–0.09	0.07	–1.21	0.23	–0.23 to 0.06
14 days	–0.12	0.05	–2.24	0.03	–0.23 to –0.01
Constant	0.21	0.07	2.97	< 0.001	0.07 to 0.35

*The variable was normalized conducting a logarithmic transformation of the data

^†^The reference category is angulation

^‡^The reference category is maxillary central incisor

^§^The reference category is absence of attachment

**The reference category is 7 days

## Discussion

The objective of this study was to investigate the predictability of OTM in full permanent dentition when the treatment had been performed in one-step exclusively with Invisalign clear aligners. A key finding derived from this study was that all teeth showed a significant difference between the planned movement and the achieved one. When that amount is interpreted in the light of clinically relevance, the lack of correction was evident in angular movements in all groups of teeth (greater than 2°). On the contrary, focusing on linear movements, it was limited to the linear buccal/lingual translation of mandibular second molars (greater than 0.5 mm).

To the best of our knowledge, our study presents several differences in materials, methodology and design compared to other published papers. At first, the sample size was much larger. We collected 79 homogeneous patients in full permanent dentition, while in other studies the sample comprised no more than 38 participants [8, 13, 24], therefore lower than recommended by the statistical power calculation of this study. In addition, our study is the first in which the predictability of OTM is evaluated along with the effect of the presence and shape of attachments and the aligner change regime.

Almost all the previous studies analyzing the efficacy of clear aligners have used percentage to describe their results [8, 11, 13]. Reporting percentage change gives the results of a trial in clinically terms immediately accessible to patients and clinicians alike. However, percentage change from baseline has been demonstrated to be statistically inefficient [32]. Finally, previously published papers do not provide any information on the real amount of analyzed movements and do not link the increase of the amount of prescribed movements with the lack of correction. This is the main reason why we decided to consider raw numerical data related to tooth position and to prescribed movements.

Regarding types of movements, the results of the present study confirm previous reports indicating tipping and torque movements as the most difficult to control with aligners and the loss of information increases moving toward the distal portion of the aligner [13, 33]. For most of the teeth angulation and inclination (mesio-distal tipping and bucco-lingual inclination), prescriptions resulted in an overexpression of those movements, with the worst conditions revealed for first and second molars on both arches. Similar results were obtained by Goh et al. [33] for the lower first molar only. Differences among the studies could be related to different applied methodologies for data collection, however, the number of aligners and treatment time of the study groups considered is comparable so careful monitoring of the vestibular torque prescription for these dental elements is strongly recommended. Furthermore, as described by Al-Nadawi et al. and Deregibus et al. [34, 35] these results may be due to the flexibility of the material and to the occlusal forces, so as well as it happens in fixed orthodontics, overcorrection should be added to the end of the customized archwire to reduce posterior arch discrepancies [36]. According to Cattaneo et al., occlusal forces can affect OTM, and therefore, if aligners do not release enough force to overcome the resistance of the system, teeth could not achieve the planned position, precluding torque and tip control [13, 37]. It should be noted that in the analyzed sample about the 60% of the second molars of each arch, had no attachments. Considering that moving from the center to the distal extremities of the aligners, their elasticity increases, it could be speculated that the use of attachments on distal teeth can increase the stiffness of the system that resulting in beneficial results in terms of angulation and inclination movements control.

Regarding rotational movement, our results reveal that rotation of the first molars is the only movement with excellent predictability, as reported in previous articles [31, 38]. On the other hand, we found that the rotational movements of the upper lateral incisors, canines and premolars are movements that are difficult to control with aligners. Several in vivo and in vitro studies confirmed the clinical experience of the poor predictability of rotational movement of rounded shaped teeth, with maxillary canines being the most affected teeth [8, 13, 15, 39, 40]. According to some reports, canines demonstrate a mean rotational discrepancy between predicted and finally achieved movement of approximately 3.8° [41]. Our data are suggesting that when we prescribe a median rotation correction of 11° for the upper canine, we are losing 4.7°, or 0.4° for every prescribed 1°. This loss of information is important and occurs even though 87% of the upper canines in our sample have an attachment (conventional or optimized): the simplest conclusion that can be drawn is that the design of the used attachments is not adequate to control the prescribed movement.

Similar results were obtained for premolars since optimized attachments offer no advantage to control their rotation: attachments are necessary [15], but current designs do not seem to work effectively.

Despite other studies revealed lower canine rotation as one of the worst movements in terms of predictability, our study revealed that a median prescribed correction of 14.8° will result in 4.8° of loss, in other words we are losing 0.3° every 1° of planned movement. Given that 70% of the lower canines in our sample had optimized attachments, the reduced lack of correction, compared with what has been reported in other studies, indicates that these attachments should be applied to improve clinical outcome.

When analyzing linear movements in the mesio-distal direction on the upper arch, canines and laterals showed 0.4 and 0.3 mm loss for every prescribed 1 mm, respectively, while first molars lost 0.4 mm for every prescribed 1 mm. This loss could be related to the fact that the 25% of first upper molars in our sample didn’t have any attachment. Our study showed that the increase in mesio-distal linear movements in the upper arch does not affect the lack of correction. This should be considered when planning overcorrections.

In the lower arch, mesio-distal movements along the arch seem to be effective for incisors, losing 0.3 mm for every prescribed 1 mm. Canines are losing almost the half of the prescribed movement, while premolars are losing almost the entire prescribed amount of movement. Lower molars are the worst teeth to control when planning movements along the arch. As already reported for rotational movements, the presence of optimized attachments seems ineffective for lower canines and premolars mesio-distal translation movements.

Among linear movements, vertical translation represented the most challenging movement with aligners: a recent study using CBCT superimpositions demonstrated a lack of correction of about the 50% when analyzing upper and lower intrusion with Invisalign [42]. Furthermore, Blundell et al. [43] reported that, on average, the Invisalign appliance expresses 39.2% of the programmed overbite reduction when compared with the prescribed outcome in the ClinCheck^®^ software and that the deeper the overbite is pretreatment, the more challenging it may be to achieve the overbite reduction. Haouili et al. [8] analyzing the efficacy of the Invisalign system reported that the higher accuracy of incisor extrusion and molar intrusion and low accuracy of incisor intrusion and molar extrusion suggests that Invisalign is more effective in bite closure, rather than bite opening. The results of the present study about vertical movements are more encouraging for lower incisors, showing that the lack of correction is of 0.3 mm for every 1 mm of prescription therefore with better results with respect to Al-Balaa et al. and Blundell et al. However, considering upper incisors and canines and lower canines, our results are in line with what has been previously reported in the existing literature [42, 43]. In addition, the most difficult teeth to control in relation to vertical movements are premolars and molars on both arches. Accordingly, to Goh et al., and Haouili et al., vertical movements of premolars and molars are poorly expressed because of the inherent biteplane effect of clear aligners and the first molar experience of the highest masticatory loads in the dental arch [8, 44, 45].

The last movement we considered was the bucco-lingual linear translation. Our results reveal the worsening of the obtained outcome moving from the center of the aligner to its distal portions, with loss increasing from 0.1 to 0.3 mm for every prescribed 1 mm, from incisors to premolars, to 0.5 mm for every 1 mm of prescribed movement for first molars. These results are consistent with what was stated by Rossini et al. in their 2014 and 2017 reviews on efficacy of arch expansion with clear aligners [46, 47]. The mechanical behavior of the distal portion of the aligners is similar to what happen with traditional fixed appliances and in relation to the decreasing amount of force exerted by the end of an arch wire as interbracket distance and flexibility of the wire increase. Therefore, lower forces could be released resulting in less accurate movement [36, 48]. This aspect could well describe the differences observed in this study between the planned and the achieved positions of the upper and lower second molars.

The main limitations of this study are represented by a non-balanced gender distribution among the sample with a large prevalence of women and the absence of a compliance objective and reliable method of measurement: therefore, we cannot define the effect of patient compliance on the measured lack of correction. Lastly, to correctly interpret the results, another limitation must be discussed. The software used in this study can measure the position of each tooth with respect to six degrees of freedom and the overlays were performed using the landmark-based method and the “best-fit alignment” algorithm. This was the best available option due to the absence of stable anatomical structures in the models obtained by the ClinCheck^®^ software.

The above limitations could be connected as well with the reduced fitting of the regression model, built on our data. In other words, the risk factors tested in our study were not sufficient to justify full data variability. Nevertheless, some information could be obtained from the regression output, regarding some exposures which influence treatment achievement and in detail OTM.

Despite all the possible limitations, to the best of our knowledge, this is the first study using stable coordinates, external to the model itself, as a reference for measurements. Furthermore, the lack of correction data could potentially be used in the clinical setting as references for overcorrection of the treatment plans in mild-to-moderate cases, as the ones considered for the study. In fact, the specific tooth movements most likely to fail to achieve the expected increase in the real-world setting were identified. In essence, this is strictly related to tooth type and direction of movement [20, 39]. According to our analyses, we know that the PM significantly affects LC, in detail, if PM increases by 10%, we expect a 2% increase in LC, and a 9% and 15% when PM rises to 50% and 100%, respectively, but that is not all.

Furthermore, our data suggest that in general changing the aligner every 14 days will reduce the LC of the 12% with respect to change aligners every 7 days. The clinical setting is therefore advisable to change aligner every 14 days especially to control the difficult movements described in the present study. Changing every 10 days does not make any difference with respect to 7-day change when considering mild-to-moderate cases.

As reported for other techniques [49, 50], there is no perfect appliance, yet despite the limitations of the system, clinical results with aligners are satisfactory [8, 51]. Interpreting our results, we should consider that each orthodontic movement consists of several components; therefore, every result isolating a single movement should be judged as a simplification of a complex reality. Furthermore, the final stage of the virtual setup (the last graphic representation) shows an approximation of the expected therapeutic result. Despite the advancement in technology, we are still not able to transfer information related to individual biology, in terms of cellular reactivity to a defined mechanical perturbation, in these virtual setups. Therefore, we must remember that the final stage of ClinCheck is the expected final result in the virtual world and an approximation of it in the real world.

## Conclusions

Few variables reach clinical significance indicating the need for additional finishing steps in aligner treatment of mild-to-moderate cases.

The present study provides the following information:

- The amounts of movement overcorrection for mild/moderate cases were identified.
- Inclination and rotation movements are the most challenging movements to control for upper laterals and for upper and lower premolars and canines, so biomechanics research in this direction should be stressed.
- Some optimized attachments design needs to be re-evaluated in the light of the results of the present study.
- Movements of second molars are mostly unexpressed: research into the thermoplastic materials with which to control these movements needs to be reinforced.
- Aligner change regimen should be defined based on the movement that needs to be controlled during treatment: a 7-day protocol is generally sufficient for most movements, but molar torque control, lower canine and bicuspid rotation and torque control and lower molars rotation need 14 days aligner change.

In conclusion, the data obtained from this clinical study contribute greatly to the ongoing debate. It is desirable that samples concerning the treatment of severe malocclusions can be analyzed in the future.

## Data Availability

The datasets used and analyzed during the current study are available from the corresponding author on reasonable request.

## Abbreviations

OTM: Orthodontic tooth movement
FA: Facial axis
FACC: Facial axis of the clinical crown
LC: Lack of correction
mLC: Mean lack of correction
PM: Prescribed movement
mPM: Mean prescribed movement
TM: Type of movement
TG: Teeth group
ICC: Intraclass correlation coefficient
Iqr: Interquartile range
