# Correction to: Predictability of orthodontic tooth movement with aligners: effect of treatment design

**DOI:** 10.1186/s40510-023-00499-8

**Published:** 2023-10-24

**Authors:** Tommaso Castroflorio, Ambra Sedran, Simone Parrini, Francesco Garino, Matteo Reverdito, Riccardo Capuozzo, Sabrina Mutinelli, Simonas Grybauskas, Mantas Vaitiekūnas, Andrea Deregibus

**Affiliations:** 1 Department of Surgical Sciences, Dental School of the University of Torino, Via Nizza 230, 10126 Turin, Italy; 2 Poggibonsi, Italy; 3 Turin, Italy; 4 Cuneo, Italy; 5 Caserta, Italy; 6 Department of Neuroscience, School of Dentistry, Section of Pedodontics, University of Padova, Via VII Febbraio 2, 35122 Padua, Italy; 7 University of Ferrara, Vilnius, Lithuania; 8 Kaunas University of Technology, Kaunas, Lithuania

## Correction to: Progress in Orthodontics (2023) 24:2 https://doi.org/10.1186/s40510-022-00453-0

Following publication of the original article [1], the authors identified an error in two sentences in the Discussion section.

The sentences currently read:

Similar results were obtained by Goh et al. [33] for the lower first molar only. Differences among the studies could be related to the fact that patients considered by Goh et al. were treated with a maximum number of 14 aligners, therefore with simplest malocclusions with respect to the ones analyzed by our team, and to different applied methodologies for data collection.

The sentences should read:

Similar results were obtained by Goh et al. [33] for the lower first molar only. Differences among the studies could be related to different applied methodologies for data collection, however, the number of aligners and treatment time of the study groups considered is comparable so careful monitoring of the vestibular torque prescription for these dental elements is strongly recommended.

The indicated sentences in the Discussion section have been updated above and the original article [1] has been corrected.
